# Supplementary material for: Scientometric and Methodological Analysis of the Recent Literature on the Health-Related Effects of Tomato and Tomato Products
Source: Foods. 2021 Aug 17;10(8):1905. doi: 10.3390/foods10081905 (PMC8393598; doi:10.3390/foods10081905)
Supplement: Supplementary file 1 [file foods-10-01905-s001.zip › Supplementary figures.pdf]

Scientometric and methodological analysis of the recent literature on the health-related effects of tomato and tomato products

Francesca Tilesi [1], Andrea Lombardi [2] and Andrea Mazzucato [2,*]

Supplementary figures

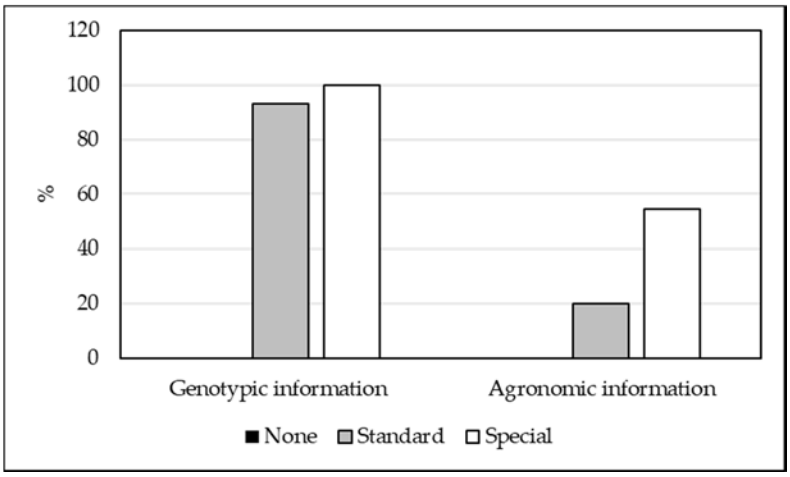

Figure S1. Percentage of studies adopting fresh material that reported genotypic and/or agronomic information about the tomato material and the growth conditions used, divided among those referring to undefined material (None), or to standard or special tomatoes.

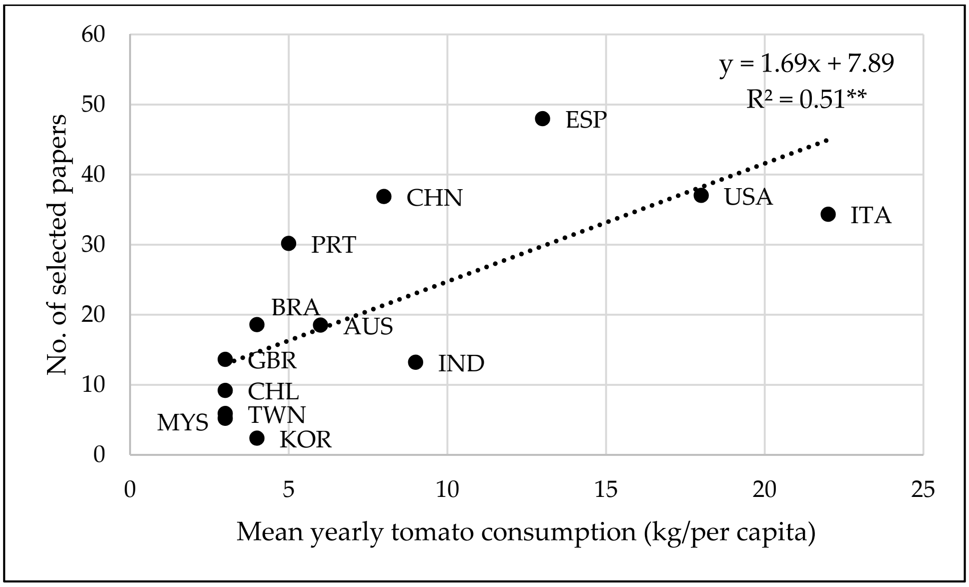

Figure S2. Linear regression of the number of selected papers and the yearly per capita tomato consumption [2] including the estimated equation and coefficient of determination. ** Indicates that the regression is significant for $P \leq 0.01$. Country symbols follow the ISO-3166 Alpha-3 code and are reported in Table 1.
